# Statistical and Machine Learning Approaches to Predict Gene Regulatory Networks From Transcriptome Datasets

**DOI:** 10.3389/fpls.2018.01770

**Published:** 2018-11-29

**Authors:** Keiichi Mochida, Satoru Koda, Komaki Inoue, Ryuei Nishii

**Affiliations:** ^1^Bioproductivity Informatics Research Team, RIKEN Center for Sustainable Resource Science, Yokohama, Japan; ^2^Microalgae Production Control Technology Laboratory, RIKEN Baton Zone Program, RIKEN Cluster for Science, Technology and Innovation Hub, Yokohama, Japan; ^3^Institute of Plant Science and Resources, Okayama University, Kurashiki, Japan; ^4^Kihara Institute for Biological Research, Yokohama City University, Yokohama, Japan; ^5^Graduate School of Mathematics, Kyushu University, Fukuoka, Japan; ^6^Institute of Mathematics for Industry, Kyushu University, Fukuoka, Japan

**Keywords:** machine learning, gene regulatory network, sparse modeling, transcriptome, time series analysis

## Abstract

Statistical and machine learning (ML)-based methods have recently advanced in construction of gene regulatory network (GRNs) based on high-throughput biological datasets. GRNs underlie almost all cellular phenomena; hence, comprehensive GRN maps are essential tools to elucidate gene function, thereby facilitating the identification and prioritization of candidate genes for functional analysis. High-throughput gene expression datasets have yielded various statistical and ML-based algorithms to infer causal relationship between genes and decipher GRNs. This review summarizes the recent advancements in the computational inference of GRNs, based on large-scale transcriptome sequencing datasets of model plants and crops. We highlight strategies to select contextual genes for GRN inference, and statistical and ML-based methods for inferring GRNs based on transcriptome datasets from plants. Furthermore, we discuss the challenges and opportunities for the elucidation of GRNs based on large-scale datasets obtained from emerging transcriptomic applications, such as from population-scale, single-cell level, and life-course transcriptome analyses.

## Introduction

Gene regulatory networks (GRNs) represent the causal relationship between genes regulating cellular functions (Barabasi and Oltvai, 2004; Blais and Dynlacht, 2005). GRNs play important roles in cellular regulatory systems such as signal transduction and transcriptional regulation, which underlie almost all cellular phenomena. Therefore, comprehensive GRN maps are essential tools to elucidate gene function, thereby facilitating interpretations of biological processes, such as cellular differentiation and response to environmental stimuli at system-level (Lopez-Maury et al., 2008), and enabling the identification and prioritization of candidates of genes for molecular regulators and biomarkers (van Dam et al., 2018).

A number of approaches have been proposed for reconstruction of GRNs based on high-throughput biological datasets. Transcriptome datasets, usually from time-series samples, have enabled us to infer gene expression networks using various statistical and machine learning ML-based algorithms (Dewey and Galas, 2010). The inferred GRNs are complementary to gene networks obtained from other types of data: transcription factor networks based on high-throughput methods to examine the interaction between transcription factors (TFs) and DNA-binding sites on target genes (Ikeuchi et al., 2018), and gene networks genetically determined using large-scale populations and mutant panels (Fuxman Bass et al., 2015; Hanson et al., 2018).

In this review, we provide an overview of recent advances in the computational inference of GRNs, based on large-scale transcriptome sequencing datasets of model plants and crops. We highlight statistical methods, including sparse modeling and machine-learning methods, for inferring GRNs based on transcriptomic datasets from plants. Furthermore, we discuss the challenges and opportunities for the elucidation of GRNs based on large-scale datasets obtained from emerging transcriptomic applications, based on population-scale, single-cell level, or life-course analyses.

## Contextual Gene Selection

Since statistical and ML-based approaches for GRN inference often have high computational complexities with high-dimensional transcriptome datasets, selection of contextual genes may be a strategy to solve the NP-hard nature (large number of genes to limited number of data points). Differentially expressed genes, including those encoding transcription factors (TFs), across spatial and temporal transcriptome datasets, form a context filter widely used to select genes for GRN inference. To predict GRNs involved in stem cell regulation in *Arabidopsis* roots using spatial and temporal transcriptome data, de Luis Balaguer et al. (2017) selected 1,625 genes, identified by their differential expression in the stem cells, and focused on 201 TF genes to infer GRNs, based on their observation of enriched GO categories, such as the regulation of transcription and TF activity in the genes. Comparing the DNA-binding capabilities between selected TFs and promoter regions of selected genes, such as DEGs and co-expressed genes, would also facilitate further narrowing down of genes that would be potentially regulated by the selected TFs in the TF network (Ni et al., 2016; Wilkins et al., 2016; Hickman et al., 2017). For example, (Wilkins et al., 2016) identified 5,447 putative target genes for 445 TFs by searching for known *cis*-regulatory motifs in open promoter regions, determined by an ATAC-seq analysis to select genes and TFs involved in a GRN that responds to environmental stimuli. Constructing an initial network by assumption-free methods, such as information theory-based methods or co-expression analysis, would be feasible to minimize false-positive edges with high computational efficiency in GRN inference, enabling us to apply statistical or ML-based methods to examine causalities between genes with respect to each local subnetwork in the initial networks (Liu et al., 2016). To predict the drought stress-responsive GRN in sunflower, Marchand et al. (2014) selected 145 genes that were co-expressed under drought stress conditions, and subsequently used a Gaussian graphical modeling method and a Random Forest method to infer the robust edges (Marchand et al., 2014). In addition to these approaches, genetics-based approaches to identify genotype-phenotype relationship can provide plausible sets of genes that are involved in a GRN. Calabrese et al. (2017) adopted an approach, integrating GWAS and co-expression network analysis, to narrow down the causal genes for bone mineral density, suggesting the feasibility of genetics-based selection of genes whose interplay underlies biological processes related to traits of interest. Through analysis of eQTL and eQTL-guided co-expression network, Basnet et al. (2016) identified candidate genes that genetically regulate the fatty acid composition in *Brassica rapa* seeds, based on *cis*- and *trans*-QTLs, detected by the eQTL analysis; this demonstrated that eQTLs can suggest a causal relationship between genes, complementary to networks inferred by computational methods.

## GRN Inference Approaches

### Statistics-Based Approaches

Since time-series datasets contain dynamic information, which assists us in understanding temporal dynamics of various biological processes, statistics-based approaches have been often applied to time-series transcriptome datasets to infer GRNs (Table 1). The Autoregressive exogenous variables (ARX) model, a kind of state-space model, is useful to describe time-varying processes observed in time-series datasets, which enable us to reconstruct a GRN in combination with sparse estimation algorithms. Fused Lasso, a sparse estimation algorithm, was employed to reconstruct GRNs with time-series expression datasets from *Escherichia coli, Mycobacterium tuberculosis*, and *Mus musculus* (Omranian et al., 2016)^1^. In a model grass *Brachypodium distachyon*, Koda et al. (2017) had formulated gene-gene temporal interactions for 3,621 periodically expressed genes, observed in a time-series RNA-Seq dataset based on ARX models, combined with a statistical sparse estimation method Group SCAD (Smoothly Clipped Absolute Deviation), a kind of L_1 regularization technique to estimate sparse GRNs, and predicted GRNs containing 2,187 genes and 3,107 directed edges. The Inferelator algorithm^2^, a kind of sparse regression approach (Greenfield et al., 2013), was also applied to infer an environmental gene regulatory influence network (EGRIN) from datasets of time-series transcriptome (RNA-Seq) and chromatin accessibility (ATAC-seq) in five tropical Asian rice cultivars to understand their physiological response to high temperature and water deficiency under agricultural field conditions (Wilkins et al., 2016). de Luis Balaguer et al. (2017) developed GENIST^3^, based on a dynamic Bayesian network algorithm, and applied it to infer GRN from cell-type specific and time-series transcriptome data of *Arabidopsis* root stem cells. Comparing its performance in GRN inference with previously published methods, the authors demonstrated that the GENIST algorithm outperformed with datasets used in DREAM 4 challenge 2.

**Table 1:** Examples of statistics-based and machine learning-based algorithms used for GRN inference in plants and other species.

Data type	Algorithm	Organism	Reference
**Statistics-based algorithms**			
Time series	Fused LASSO regression	*Escherichia coli*	Omranian et al., 2016
		*Mycobacterium tuberculosis*	
		*Mus musculus*	
	ARX model and GroupSCAD	*Brachypodium distachion*	Koda et al., 2017
	Inferelator	*Oryza sativa* (five tropical Asian rice)	Wilkins et al., 2016
	GENIST	*Arabidopsis thaliana*	de Luis Balaguer et al., 2017
Non-time series	CLR	*Zea maize*	Xiong et al., 2017
	PRC	*Saccharomyces cerevisiae*	Blum et al., 2018
**Machine learning-based algorithms**			
Various biological and experimental conditions	MinReg	*Fusarium graminearum*	Guo et al., 2016
Various tissues	GENIE3	*Zea maize*	Huang et al., 2018
Time series (spatial and temporal)	DFG	*Arabidopsis thaliana*	Varala et al., 2018

There also exist examples of statistics-based approaches to infer GRNs using non-time-series transcriptome datasets (Table 1). Xiong et al. (2017) inferred GRNs to identify key genes in the maize seed development process with RNA-Seq data from various tissues: embryos, endosperms, whole seeds, and other tissues, by Context Likelihood of Relatedness (CLR^4^) (Faith et al., 2007), which is a mutual information (MI)-based GRN inference approach (Xiong et al., 2017). They inferred gene regulatory relationship based on z-score of MI between a TF gene and a non-TF or another TF gene, and generated a GRN composed of 10,932 nodes and 48,740 edges. They also verified eight regulatory relations between TF and non-TF genes, through yeast one-hybrid (Y1H) assay, to assess TF-promoter binding, and assessed the Opaque-2 TF network, inferred in the GRN, by comparing it with previously identified regulatory network based on the results from ChIP-seq analysis and RNA-Seq analysis of its mutants. Since GRNs inferred by MI-based approach basically show undirected graph, regulatory relations between genes are usually based on their putative function, i.e., TF-encoding genes or non-TF genes. To estimate regulatory relations between genes, Blum et al. (2018) developed a GRN inference algorithm based on partial response coefficients (PRC), and assessed its performance on synthetic datasets, as well as transcriptome datasets, from gene knockout mutants of yeast (Kemmeren et al., 2014), demonstrating its superior performance for GRN inference in studies with large-scale knockout mutant resources (Blum et al., 2018).

### Machine Learning-Based Approaches

Machine learning, an area of computer science that offers data-driven prediction, has attracted wide attention for its various applications in modern biology (Camacho et al., 2018; Webb, 2018), besides putting forth its strength in GRN inference (Table 1). Guo et al. (2016) applied the MinReg algorithm^5^, based on a derivative of Bayesian networks, and a greedy algorithm, to infer the global GRN in *Fusarium graminearum* with 27 (9 experiments with three biological replicates) and 166 transcriptome datasets retrieved from the PLEXDB (Guo et al., 2016). They identified 968 candidates of regulators and represented a subnetwork for a regulatory gene *FAC1* by superimposing information from its protein–protein interaction (PPI) network and the differentially expressed genes of its mutant in *F. graminearum*. GENIE3^6^, a tree-based ML algorithm (Huynh-Thu et al., 2010), has been widely employed in recent GRN inference studies with both static and dynamic transcriptome data from various species (Banf and Rhee, 2017; Desai et al., 2017; Redekar et al., 2017). Huang et al. (2018) applied GENIE3 to infer GRNs with over 1,000 publicly available RNA-Seq data from various tissues such as leaf, root, shoot, apical meristem, and seed, and created four tissue-specific GRNs. They validated the predicted regulatory networks for transcription factors KN1 (KNOTTED1), FEA4 (fasciated ear4), and O2 (Opaque2), by using publicly available ChIP-seq datasets. Varala et al. (2018) applied dynamic factor graph (DFG) models (Mirowski and LeCun, 2009) to a fine-scale time-series transcriptome in response to nitrogen supply in *Arabidopsis* shoots and roots, and illustrated a GRN composed of nitrogen responsive TF and non-TF genes (Varala et al., 2018). They validated the predicted regulatory networks for transcription factors CRF4, SNZ, and CDF1, which showed early N-response in shoots and roots, by using the TARGET (Transient Assay Reporting Genome-wide Effects of Transcription factors) method, and demonstrated that five key genes involved in N uptake and assimilation were included in the predicted and validated targets of these three TFs (Bargmann et al., 2013). These examples suggested that ML-based approaches provide opportunities to reconstruct GRNs from various types of transcriptome datasets, thereby assisting the identification of key TF-genes involved in cellular systems related to various biological functions in plants.

### Combined Approaches

Combinatorial use of multiple algorithms could be a promising strategy for GRN inference (Marbach et al., 2012; Table 2). Foo et al. (2018) employed three different algorithms, Inferelator, TIGRESS (Trustful Inference of Gene REgulation with Stability Selection^7^) (Haury et al., 2012), and GENIE3, to infer GRNs involved in defense response of *Arabidopsis* with its microarray-based time-series transcriptome data (Foo et al., 2018), and verified a particular subnetwork using Y1H assay, information from an *Arabidopsis* cistrome map, and gene expression profiles from overexpressors of a related gene. Redekar et al. (2017) used five different algorithms, ARACNE^8^ (Margolin et al., 2006), GENIE3 (Huynh-Thu et al., 2010), TIGRESS (Haury et al., 2012), partial correlation (GeneTS^9^) (Schafer and Strimmer, 2005), and CLR (Faith et al., 2007), to infer the GRNs between TFs and co-expressed modules for seed development in soybean^10^, based on 60 RNA-Seq datasets (three biological replicates, five stages of developing seeds, and four experimental lines), and evaluated the resultant GRNs by comparative analysis with published GRNs of *Arabidopsis* (Redekar et al., 2017)^10^. Banf and Rhee developed a novel GRN inference strategy called GRACE (Gene Regulatory network inference ACcuracy Enhancement^11^), which generates GRNs through multiple steps to integrate various knowledge related to the regulation of gene expression: initial network prediction from gene expression data using a random forest regression model and integrating information related to gene regulation, subsequent network module extraction by meta-network construction based on information of functionally related genes, and further selection of regulatory links using ensembles of Markov Random Fields (Banf and Rhee, 2017). To infer the developmental GRN in *Arabidopsis*, the authors incorporated conserved sequence information in its promoter regions and experimentally determined *cis*-motifs for TFs, together with gene expression data from 83 tissues and stages, and obtained an initial GRN containing 325 regulators, 4,305 targets, and 10,098 links. To enhance confidence of the initially predicted GRN, the authors integrated knowledge from various information resources such as AraNet^12^, ATRM (Arabidopsis Transcriptional Regulatory Map^13^), SUBA3^14^, and AraCyc^15^, and demonstrated its potential to produce high-confidence regulatory networks, thereby suggesting a benefit of integration of multiple clues from various information resources to improve accuracy of the GRNs.

**Table 2:** Examples of combined approaches for GRN inference in plants and other species.

Data type	Algorithm	Organisms	Reference
Time series	Inferelator, TIGRESS, and GENIE3	*Arabidopsis thaliana*	Foo et al., 2018
	ARACNE, GENIE3, TIGRESS, Partial correlation, and CLR	*Glycine max*	Redekar et al., 2017
Time series (development)	GRACE (Random forest and ensembles of Markov Random Fields)	*Arabidopsis thaliana*	Banf and Rhee, 2017
		*Drosophila melanogaster*	

## GRNs With Emerging Applications

In terms of recent advances in both resolution and throughput to acquire genome and transcriptome datasets (Reuter et al., 2015), and computational methodologies to analyze the datasets, GRNs have yielded various applications which allow us deeper understanding of cellular systems at population, life-course and single-cell level. Here, we highlight emerging applications of these approaches, through GRN reconstruction, from these three specific aspects.

### Population Transcriptomics for GRN Construction

Population-scale transcriptome sequencing enables us to shed light on molecular consequences of regulatory variations in complex traits. Through transcriptome sequencing across mapping populations, eQTL analysis has been widely used to identify *cis*- and *trans*-QTLs, and reconstruct regulatory networks to mine genetic factors that determine various traits, including agronomic traits of crop species (Albert et al., 2018; Galpaz et al., 2018; Wang et al., 2018; Zhang et al., 2018). Moreover, a transcriptome-wide association study (TWAS) was proposed to identify associations between gene expression and traits (Gusev et al., 2016), and has recently been applied to construct GRNs. For example, integrating genome and transcriptome data of whole blood RNA-Seq samples across 3,072 unrelated individuals, Luijk et al. (2018) constructed a GRN that suggests 49 regulatory genes that affect transcriptional changes of their downstream genes. Moreover, population-scale transcriptome sequencing across multiple tissue types, have been applied to reconstruct GRNs through integration with other resources on molecular networks, such as PPI and TF motifs, to reveal tissue-specific gene regulation (Sonawane et al., 2017).

### Spatial-Temporal GRNs at Single-Cell Level

High-throughput sequencing applications at single-cell level have rapidly emerged, and enabled us to decipher GRNs underlying cellular heterogeneity (Liu and Trapnell, 2016; Libault et al., 2017; Dasgupta et al., 2018; Fiers et al., 2018). For GRN inference from single-cell transcriptome datasets, several computational algorithms have recently been developed. Chan et al. (2017) developed an algorithm, PIDC, which identifies regulatory relations between genes based on partial information decomposition (PID), and is applied to infer GRNs from single cell-qPCR datasets. SCENIC^16^ constructs GRNs and identifies cell-status based on scRNA-Seq data, which uses GENIE3 to predict TF targets based on co-expression, RcisTarget to assess TF-motif enrichment, and AUCell to assess regulon activities in each cell; it was recently applied to GRN analysis in a single-cell transcriptome from adult fly brain sampled across its lifespan (Davie et al., 2018). Although, till date, there are only a small number of scRNA-Seq datasets from higher plant species (Perroud et al., 2018), single-cell level high-throughput data in plants, and GRNs based on such datasets, will provide invaluable resource to facilitate in-depth elucidation of various cellular systems in plants (Efroni and Birnbaum, 2016).

### GRNs Throughout Life-Course

Longitudinal transcriptome study provides insights into the trajectory of GRNs, underlying the biological phenomena throughout life-span/life-course, such as aging and phenology. Through a longitudinal transcriptome analysis of short-lived killifish, *Nothobranchius furzeri*, Baumgart et al. (2016) identified mitochondrial respiratory chain complex I genes as the hub in a co-expressed gene expression module that negatively correlated with its lifespan. For crop improvements, trajectories of physiological states, resulting from interaction between genetic and environmental factors, often influence the phenotypes of eventual agronomic traits; longitudinal study of cellular networks provides clues to identify gene-environment interactions associated with the phenotypic changes in crops (Mochida et al., 2015; Sun and Dinneny, 2018). Through construction of an integrated atlas of gene expression and regulatory networks in developing maize, Walley et al. (2016) demonstrated that integration of transcriptome, proteome, and phospho-proteome data can improve GRN inference. In tropical rice, as introduced in the previous sections, integrating time-series datasets of transcriptome, nucleosome-free chromatin from ATAC-seq, and known *cis*-motifs for TFs from five tropical rice cultivars under controlled and agricultural field conditions, Wilkins et al. (2016) constructed GRNs that represent relationships between the timing and gene expression in response to environmental changes. These examples from staple crops illuminate that combinatorial use of multiple omics data is a promising approach to improve the performance of GRN inference, as well as to mine better clues to improve agronomically important traits of crops under field conditions.

## Conclusion and Perspectives

In the last few years, approaches to reconstruct GRNs have advanced by synergistic innovation of high-throughput sequencing and computational techniques; GRNs have played crucial roles to elucidate cellular systems and identify key genes that manipulate cellular functions. A lot of statistical- and ML-based approaches have been proposed and applied to infer GRNs based on transcriptome datasets; these have contributed to identify regulatory relationships of genes involved in various biological phenomena in plants. Coupled with applications recently developed in high-throughput sequencing, GRNs dramatically improve their resolution with emerging aspects of transcriptomics, such as across accessions/individuals, cell types, and life stages, each of which provides opportunities to address challenges for these emerging areas in plant science.

Integration of GRNs and other networks, such as epigenetic, PPI, and metabolic networks, provides clues to identify molecular relations that function as interfaces, and will provide new insights into *trans*-omics networks across multiple omics layers (Yugi et al., 2016). ML has provided algorithms to find useful patterns from large and heterogeneous (unstructured) data, acquired through multiple high-throughput techniques (Ma et al., 2014; May, 2014; McCue and McCoy, 2017). Recently, ML-based approaches have been applied to extract features associated with cellular states and responses from high-throughput data, including transcriptomic and epigenomic data, and develop computational models that classify the cellular states and responses in applications such as precision oncology and drug development (Aliper et al., 2016; Malta et al., 2018). In plant science, ML-based integrative analysis of large-scale data from multiple omics spectra, such as genomic variations and molecular networks, as well as high-throughput phenomics, will enable us to decipher complex cellular systems and figure out molecular features associated with quantitative traits in plants and crops, and apply the results to design traits through optimizing GRNs in crop breeding. From the perspective of ML in GRN study, it will offer us algorithms not only for GRN inference but also for feature extraction across multi-dimensional datasets from various high-throughput experimental techniques.

## Author Contributions

KM, SK, KI, and RN conceived the study, performed the research, and wrote the manuscript.

## Conflict of Interest Statement

The authors declare that the research was conducted in the absence of any commercial or financial relationships that could be construed as a potential conflict of interest.

## Footnotes

1 https://github.com/omranian/inference-of-GRN-using-Fused-LASSO

2 https://sites.google.com/a/nyu.edu/inferelator/home

3 https://github.com/madeluis/GENIST

4 http://m3d.mssm.edu/network_inference.html

5 http://www.cs.huji.ac.il/labs/compbio/minreg/

6 https://bioconductor.org/packages/release/bioc/html/GENIE3.html

7 http://projets.cbio.mines-paristech.fr/tildeahaury/svn/dream5/html/index.html

8 http://califano.c2b2.columbia.edu/aracne/

9 https://bioconductor.org/packages/2.1/bioc/html/GeneTS.html

10 https://lilabatvt.github.io/LPANetwork/

11 https://github.com/mbanf/GRACE

12 http://www.functionalnet.org/aranet/about.html

13 http://atrm.cbi.pku.edu.cn/

14 http://suba.live/

15 https://www.plantcyc.org/databases/aracyc/15.0

16 https://github.com/aertslab/SCENIC
